# SOLO AND PARENT–CHILD DYAD CAREGIVING FOR OLDER ADULTS: IMPLICATIONS FOR CAREGIVER WELL-BEING

**DOI:** 10.1093/geroni/igad104.2226

**Published:** 2023-12-21

**Authors:** Maria Kurth, Kelly Chandler

**Affiliations:** Penn State University, University Park, Pennsylvania, United States; Oregon State University, Corvallis, Oregon, United States

## Abstract

Family caregiving for an older adult is predominantly studied as a solo endeavor (NIA, 2022), overlooking care by family subsystems (see Keith, 1995) and youth (Armstrong-Carter et al., 2021). Informed by family systems theory (Cox & Paley, 1997), we analyzed data from the 2019 AARP caregiver survey (NAC & AARP, 2021) to examine demographics, experiences, and outcomes between solo (96%) and parent-child ( < 18) dyad (4%) caregivers of an adult 65+ (N = 921). Adult caregivers were mostly female (59%), non-Hispanic White (63%), and cared for an old-old recipient (Mage = 81. 97, SD = 8.93). On average, caregivers were in midlife (Mage = 60.98, SD = 14.86, range: 19-93). Caregivers with child assistance were more likely to be Hispanic, X2(1) = 6.34, p < .05, employed, X2(1) = 10.87, p < .01, younger, t(46) = 8.79, p < .001, and cared for a younger recipient, t(45) = 3.40, p <.01. They were more likely to receive unpaid help, X2(1) = 28.02, p < .001, care for their father, X2(1) = 5.15, p < .05, and less likely to provide care to a spouse, X2(1) = 5.91, p <. 05, and identify as the primary caregiver, X2(1) = 6.42, p < .05. Preliminary ANCOVA results suggested parent-child dyad caregivers had less emotional stress F(1) = 5.64, p < .05; groups were comparable for financial strain and sense of purpose. Results add to the family caregiving literature by highlighting the experiences of parent-child caregiving dyads, which might buffer against caregiving stress.

